# Patients’ experiences of a patient-centred polypharmacy medication review intervention: a mixed-methods study

**DOI:** 10.3399/BJGP.2025.0052

**Published:** 2025-11-18

**Authors:** Lorna J Duncan, Deborah McCahon, Barbara Caddick, Roxanne M Parslow, Katrina Turner, Carolyn A Chew-Graham, Bruce Guthrie, Rupert A Payne

**Affiliations:** 1 Centre for Academic Primary Care, Population Health Sciences, Bristol Medical School, University of Bristol, Bristol, UK; 2 School of Medicine, Keele University, Keele, UK; 3 Advanced Care Research Centre, University of Edinburgh, Edinburgh, UK; 4 Exeter Collaboration for Academic Primary Care, University of Exeter, Exeter, UK

**Keywords:** clinical trial, general practice, medicines optimisation, polypharmacy, decision making, shared, qualitative evaluation

## Abstract

**Background:**

People prescribed multiple medications need regular review to optimise medication and improve health outcomes. Although the most effective method to improve inappropriate polypharmacy remains unclear, medication reviews incorporating patient-centred care and shared decision making are believed to be key to achieving optimal outcomes.

**Aim:**

To explore patients’ experiences of an intervention to deliver patient-centred polypharmacy medication review in primary care.

**Design and setting:**

A mixed-methods process evaluation was undertaken within the Improving Medicines use in People with Polypharmacy in Primary Care (IMPPP) randomised controlled trial conducted in the Bristol and West Midlands regions of England.

**Method:**

Participants receiving the intervention were invited to complete a patient experience survey. Additionally, participants were purposively sampled and invited to participate in an interview and/or audio-recording of their medication review, to explore views and experiences.

**Results:**

Survey response rate was 72.5% (*n* = 556/767); 28 patients were interviewed, 27 reviews were recorded. Overall, 73.2% (*n* = 407) were satisfied with their review, strongly associated with shared decision making, and 79.9% (*n* = 444) expressed satisfaction with how the review was delivered (primarily pharmacist-led telephone consultations). Most audio-recordings of reviews demonstrated collaborative decision making. Patients valued reviews when they felt well-informed, prepared, and received clear follow-up. Pharmacist-led delivery was acceptable, although unfamiliarity with the reviewer and concerns about prescribing authority were perceived negatively.

**Conclusion:**

Participants were satisfied with their review, although this may be contingent on preparation and support. Findings highlight the importance of communication throughout, and how the clinician role and familiarity shape patient experience. A person-centred review approach has the potential to improve patient experience, satisfaction, and engagement.

## How this fits in

Medication reviews are a central part of the process of medication optimisation, and a key policy strategy aiming to improve safe and effective prescribing and health outcomes, despite a relative lack of evidence for their effectiveness. Holistic patient-centred care can help align treatment with patients’ values and preferences, improve patient satisfaction, enhance treatment, and improve outcomes, but is not always achieved in current practice. The Improving Medicines use in People with Polypharmacy in Primary Care model of medication review delivery, including appropriate clinician training and pre-review patient preparation, achieves patient-centred care and good patient satisfaction, and may have the potential to enhance patient engagement and improve experience of care.

## Introduction

Polypharmacy is the use of multiple medications by a single individual, and is common among older adults or those with multiple long-term conditions.^1^ Although polypharmacy can be appropriate, benefits are not always realised and there is the potential for drug-related harms.^2^ Regular medication reviews, involving the patient and a prescriber, may offer opportunities to optimise medicines’ use and consequent health outcomes. Reviews should involve a comprehensive assessment to identify therapeutic priorities, discuss best available evidence, and reach agreement on what medicines to use.^3,4^

Although the most effective method to optimise medicines in people with potentially problematic polypharmacy remains uncertain, integrating holistic, patient-centred care (that is, focusing on the patient’s needs, preferences, and values) and shared decision making (that is, involving collaborative discussion between practitioner and patient) within the process may have value. Within the context of long-term condition management, these approaches may help patients and practitioners reach agreement on treatment plans that align with patient preferences, improve patient satisfaction, and enhance treatment adherence.^5,6^ However, research suggests that patients often feel unprepared or are not offered an opportunity to actively engage during medication reviews.^7^ Furthermore, many healthcare professionals lack the necessary skills for conducting reviews tailored to individual patients’ needs.^7–9^

The Improving Medicines use in People with Polypharmacy in Primary Care (IMPPP) trial evaluated a complex intervention delivering person-centred medication reviews to improve inappropriate polypharmacy in English general practice.^10^ As patient experience is an indicator of quality of care and positively associated with safety and clinical effectiveness,^11^ the aim of the current analysis was to explore patients’ experiences of medication review in the intervention arm.

## Method

The IMPPP trial was a cluster randomised two-arm parallel design, conducted in English general practices, comparing a complex intervention against usual care. The conceptual framework incorporated principles of pharmaceutical care, shared decision making, behaviour change theory, and integrated primary care practice. The protocol has been published.^10^

Thirty-seven practices (19 intervention) participated in the main trial, and a further five practices (three intervention) participated in an external pilot. The primary outcome was rate of potentially inappropriate prescribing (PIP; defined based on clinical code-based rules, as listed in the trial protocol),^10^ with secondary outcomes including other prescribing measures (for example, adherence and treatment burden), health service use, and mortality. A mixed-methods process evaluation, including a post-intervention patient experience survey, qualitative interviews, and audio-recordings of medication reviews, was included to understand the experiences of patients and clinicians. Clinicians’ perceptions of the intervention are reported elsewhere.^12^ The main trial effectiveness results are not yet published.

### Trial population, eligibility criteria, and recruitment

Potentially eligible trial participants aged ≥18 years prescribed ≥5 regular medicines, with at least one indicator of PIP, were identified pre-randomisation via an automated search of their electronic primary healthcare records. PIP indicators were defined using a set of clinical code-based rules, incorporating prescribing, diagnostic, and test codes as detailed in the trial protocol.^10^ Exclusion criteria, applied by a clinician at each site, included those deemed clinically inappropriate for contact (for example, end-of-life care) or unable to complete surveys without support. Eligible patients were invited by post before randomisation. Individuals interested in participating completed a postal consent form and a baseline survey. Each practice was asked to deliver the medication review to up to 50 consented patients over a 6-month period.

### Intervention description

The intervention involved a structured process for medication review with a comprehensive clinical encounter aimed at improving the clinical effectiveness and safety of the medication regimen (for example, through stopping or starting drugs, dose adjustment, and appropriate monitoring) tailored to the individual’s clinical circumstances (such as, age, frailty, and comorbidities) and personal values and preferences. The process comprised: automated case-finding; a pharmacist-conducted case-note review; an interprofessional collaborative discussion between pharmacist and GP; a patient-facing medication review (led by either the pharmacist or GP); and subsequent follow-up as deemed clinically necessary.

This was supported by intervention components designed to enhance engagement, including financial incentives for practices, performance feedback, and clinician training. Participating GPs and clinical pharmacists completed a comprehensive training programme covering topics essential for patient-centred medication reviews, including key consultation and communication skills (for example, relating to shared decision making).

To enhance patient engagement in the review, participating practices were asked to provide patients with a pre-review leaflet, containing information about the purpose of a review and how to prepare for it, alongside a list of their current medicines (see Supplementary Information S1).

### Audio-recording of reviews and patient interviews

On providing consent at baseline, participants could optionally choose to participate in an audio-recording of their review and/or a post-review interview (see Supplementary Figure S1). Those individuals were purposely sampled to maximise variation in age, sex, and education, with no specific exclusion criteria applied. Invitations to participate in audio-recordings were mailed out before the review.

Selection and invitation for interview was undertaken post-review without knowledge of post-review survey completion. Interview times considered participant preference and included weekends and evenings to maximise inclusivity. Additional explicit consent for these activities was obtained. Interviews were conducted via telephone or video-call by two experienced qualitative researchers (the first and fourth authors) between February 2021 and June 2023. Reviews and interviews were audio-recorded and transcribed verbatim.

Interviews were guided by a topic guide developed in collaboration with lay IMPPP study advisors and revised as data collection progressed to reflect early findings (see Supplementary Information S2). During interviews, participants were invited to share their experiences of the review, including their expectations and preparations before the review. Data collection continued until no new insights or themes were identified.

Analysis of interview and audio-recorded reviews was ongoing and iterative, facilitated using NVivo (version 11) software. Thematic analysis was undertaken, employing deductive and inductive approaches to scrutinise the data for anticipated and emergent themes, and contextual factors that enhanced understanding of the survey findings. Anticipated themes included concepts in the patient experience survey and elements of shared decision-making models identified by Makoul and Clayman,^13^ summarised in the SHARE (seek, help, assess, reach, evaluate) approach.^14^ Analysis was led by the first author, with a subset of transcripts independently read and coded by the joint first author. A coding framework was refined through consensus, and key themes discussed with the wider team (the fourth, fifth, and sixth authors) to ensure credibility and external validity.

### Survey

Patient experience surveys were administered via post or electronically (in accordance with patient preferences expressed at recruitment) within 4 weeks of receiving the patient-facing review. Pre-paid envelopes were provided to encourage return of completed surveys. The survey (see Supplementary Information S3) captured the characteristics of the review (reviewer’s profession, duration, how the review was delivered, and medication changes made); overall satisfaction with the review and confidence in the reviewer (5-point Likert scales); perceptions of the extent to which the reviewer involved the patient in shared decision making (9-item Shared Decision Making Questionnaire [SDM-Q-9]^15^ and three components of the CollaboRATE scale);^16^ alongside patient-centredness, and processes and outcomes from the patient’s perspective (four items from the Consultation And Relational Empathy measure^17^ and four items from the Patient Assessment of Chronic Illness Care measure).^18^ Patient sociodemographics were captured separately pre-randomisation.^10^

Survey results were reported according to relevant patient experience themes used in the qualitative analysis of interviews using descriptive statistics. Responses to Likert scales were recoded where appropriate to facilitate analysis and interpretation. Associations between responder satisfaction and sociodemographic factors, review context, and shared decision making were examined using mixed-effects logistic regression (see Supplementary Table S1). Analyses were conducted using Stata (version 18). Survey results using original coding and including figures for missing data, grouped by instrument, are included in Supplementary Table S2.

Findings from the survey, audio-recordings, and interviews were triangulated to provide context and a comprehensive understanding of patient experiences.

## Results

### Participant characteristics

A total of 767 participants (36 pilot study and 731 main trial) receiving the patient-facing review were invited to complete the experience survey (February 2021–April 2023), with 471 (61.4%) opting for a postal (rather than an online) survey, and 556 (72.5%) responding across all intervention practices. The median age of responders was 73 (interquartile range 66–79) years, 259 (46.6%) were female, and 531 (95.5%) identified as White British ethnicity (Table 1).

Survey responders and non-responders were broadly comparable, although responders tended to be older, retired, less socioeconomically deprived, hold higher educational qualifications (Table 1), and were more likely to have requested a paper survey (*n* = 410, 73.7% versus *n* = 146, 26.3% online survey).

**Table 1.** Survey responder characteristics (*N* = 767)^a^

Characteristic	Survey non-responders (*N* = 211), *n* (%)	Survey responders (*N* = 556), *n* (%)	Interview participants (*N* = 28), *n* (%)
**Age (years)**			
≤50	18 (8.5)	16 (2.9)	2 (7.1)
51-65	53 (25.1)	111 (20.0)	5 (17.9)
66-80	106 (50.2)	325 (58.5)	18 (64.3)
≥81	34 (16.1)	104 (18.7)	3 (10.7)
**Sex**			
Male	100 (47.4)	297 (53.4)	17 (60.7)
Female	111 (52.6)	259 (46.6)	11 (39.3)
**Index of Multiple Deprivation quintile**			
1 (most deprived)	32 (15.2)	58 (10.4)	1 (3.6)
2	47 (22.3)	98 (17.6)	7 (25.0)
3	39 (18.5)	101 (18.2)	4 (14.3)
4	53 (25.1)	153 (27.5)	6 (21.4)
5 (least deprived)	39 (18.5)	145 (26.1)	10 (35.7)
**Education level**			
No educational qualifications	62 (29.4)	123 (22.1)	8 (28.6)
GCSE, A -Levels, or equivalent	86 (40.8)	160 (28.8)	4 (14.3)
University degree or higher	25 (11.8)	110 (19.8)	8 (28.6)
Other	19 (9.0)	118 (21.2)	5 (17.9)
**Employment**			
Full or part-time work	39 (18.5)	76 (13.7)	6 (21.4)
Retired from work	117 (55.5)	386 (69.4)	17 (60.7)
Other	38 (18.0)	58 (10.4)	3 (10.7)
**Living situation**			
Own home (rented or owned)	187 (88.6)	506 (91.0)	27 (96.4)
Other	17 (8.1)	34 (6.1)	1 (3.6)
**Live alone**			
No	137 (64.9)	384 (69.1)	20 (71.4)
Yes	61 (28.9)	148 (26.6)	7 (25.0)
**Ethnicity**			
White British	190 (90.0)	531 (95.5)	28 (100)
Other ethnic group	14 (6.6)	12 (2.2)	0 (0.0)

^a^Percentages based on participant total for each column; percentages do not sum to 100% owing to missing data. GCSE = General Certificate of Secondary Education.

Interviews were conducted with 28 participants (nine pilot and 19 main trial) from nine practices. All interviewees were of White British ethnicity, 17 were male, and age ranged from 47–90 years. Interview duration ranged from 12–53 mins (mean 29 mins).

Twenty-seven reviews were audio-recorded (10 pilot and 17 main trial) within eight practices, including 19 males, and age ranged from 51–92 years.

### Overall satisfaction and perceptions of patient-centred care

Overall, 407, 73.2% of survey responders were satisfied with their medication review (Table 2), with no evidence of an association between satisfaction and sociodemographic factors (see Supplementary Table S1). Most responders reported receiving patient-centred care (Table 2). Many responders rated the reviewer as good–excellent at ‘being interested in them as a whole person’ (*n* = 405, 72.8%), ‘understanding their concerns’ (*n* = 408, 73.4%), and ‘helping them take control’ (*n* = 399, 71.8%). Views were more mixed about reviewers’ consideration of responders’ ‘values and traditions’ (*n* = 289, 52.0% yes and *n* = 237, 42.6% no/not sure) or ‘treatment plans aligning with daily life’ (*n* = 257, 46.2% yes and *n* = 271, 48.7% no/not sure).

**Table 2.** Overall satisfaction and perceptions of patient-centred care (*N* = 556)^a^

Survey question	*n* (%)
**Satisfaction**	
Overall satisfaction with review	
Dissatisfied or very dissatisfied	50 (9.0)
Neither dissatisfied nor satisfied	88 (15.8)
Satisfied or very satisfied	407 (73.2)
**Patient-centred care**	
Interest in patient as a whole person (CARE instrument)	
Poor or fair	114 (20.5)
Good	120 (21.6)
Very good or excellent	285 (51.3)
Fully understanding patient’s concerns (CARE instrument)	
Poor or fair	101 (18.2)
Good	128 (23.0)
Very good or excellent	280 (50.4)
Helping patient take control (CARE instrument)	
Poor or fair	101 (18.2)
Good	120 (21.6)
Very good or excellent	279 (50.2)
Reviewer considered patient’s values and traditions (PACIC instrument)	
No	88 (15.8)
Yes	289 (52.0)
Not sure	149 (26.8)
Treatment plan that aligned with daily life (PACIC instrument)	
No	189 (34.0)
Yes	257 (46.2)
Not sure	82 (14.7)

^a^Percentages based on total survey responders; percentages do not sum to 100% owing to missing data. CARE = Consultation And Relational Empathy measure. PACIC = Patient Assessment of Chronic Illness Care measure.

**Figure 1.**
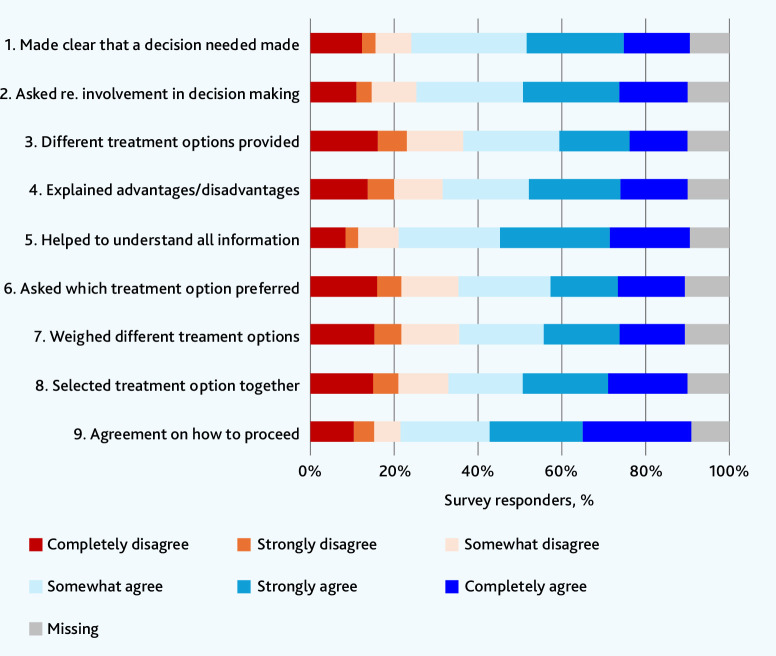
Patient-reported shared decision making (SDM-Q-9 instrument) original wording of questions is provided in Supplementary Information S3. re. = regarding. SDM-Q-9 = 9-item Shared Decision Making Questionnaire.

Responses to the SDM-Q-9 instrument are reported in Figure 1. Two-thirds (*n* = 365, 65.6%) of survey responders rated shared decision making above the midpoint of the total SDM-Q-9 score suggesting that patients felt their reviewer engaged them in treatment-related decision making. After adjustment for sociodemographic factors and aspects of the review context, there was strong evidence (*P*<0.001) that the likelihood of being satisfied was positively associated with a perception of shared decision making (odds ratio [OR] 2.16, 95% confidence interval [CI] = 1.58 to 2.94 per quintile increase in SDM-Q-9 score; Supplementary Table S1).

Analysis of the interviews and audio-recordings of reviews provided insights into:

- why patient expectations were met (or not);
- how patient views and experiences were affected by how the review was delivered;
- the patient–practitioner relationship; and
- key elements of shared decision making (for example, seeking patient participation, information provision, discussion of patient priorities, agreement on decisions, and summarising/follow-up).

These themes are discussed below using both qualitative and survey data.

### Patient expectations

Many interviewees valued their review because it enabled them to gain a better understanding of their medications, receive reassurance that their treatment was effective and appropriate, and/or discuss changes to medications. Some, however, described the review as unnecessary because their medication regimen was simple, easy to manage, and working well for them. A few described the review as impersonal or unhelpful, seeing it as a procedural requirement that offered nothing new:

*‘If you see a marked change in things, then it’s worth it. If it’s ... just a lip service review … to meet a quota, then it’s pointless … they could be more constructive with their time, putting it into other things ... for other people.’* (Pt25)

There was evidence that the trial ‘pre-review leaflet’ was not implemented as planned in some practices and this had a negative impact on patient experience. Some interviewees who had not received the leaflet expressed a desire for information in advance of the review to improve understanding of the process and support active involvement, including information regarding what issues might be considered during the review or provision of a list of their medicines to act as an *aide memoire*. Prior information and time to reflect and formulate questions in advance was important for ensuring the review was productive and beneficial:

*‘It would have been nice if I had a form beforehand that might have said the format of the discussion … so I could have written down a few things to remind myself.’* (Pt13)

### How reviews were delivered

Table 3 reports survey findings related to how the reviews were delivered. Electronic health records documented mode of review delivery for 465 survey responders, of which 347 (74.6%) were telephone consultations. In total, 444 (79.9%) responders reported they were happy with where the review took place. Responders reported 51.3% (*n* = 285) and 22.3% (*n* = 124) of reviews lasting 10–20 min and >20 min, respectively, with 453 (81.5%) considering the time they had adequate. The odds of being satisfied with the review were strongly associated with the duration (OR 32.2, 95% CI = 16.8 to 61.4) and location (OR 13.8, 95% CI = 8.2 to 23.0) of the review being considered acceptable (see Supplementary Table S1).

**Table 3.** How the medication review was delivered (*N* = 556)^a^

Characteristic	*n* (%)
**Duration of review, min**	
<10	130 (23.4)
10 to 20	285 (51.3)
>20	124 (22.3)
**Was the IMPPP medication review long enough?**	
No	33 (5.9)
Yes	453 (81.5)
Not sure	54 (9.7)
**Happy with where review appointment took place?**	
No	54 (9.7)
Yes	444 (79.9)
Not sure	37 (6.7)
**Professional conducting the review**	
GP	71 (12.8)
Pharmacist	381 (68.5)
Don’t know	77 (13.8)
**Previously received care from the reviewer**	
No	413 (74.3)
Yes	126 (22.7)
**Level of confidence in reviewer**	
Not at all or a little confident	96 (17.3)
Reasonably confident	144 (25.9)
Mostly or very confident	303 (54.5)

^a^Percentages based on total survey responders; percentages do not sum to 100% owing to missing data. IMPPP = Improving Medicines use in People with Polypharmacy in Primary Care.

There were 21 interviewees who indicated the review was conducted via telephone, with views on this mode of delivery varying. Several felt that telephone was more convenient than attending the practice and did not see a need for a discussion focused on medication to be conducted in-person:

*‘Over the phone … saves me a lot of time and energy. I don't think the outcome is any different.’* (Pt3)

Others expressed a strong preference for in-person reviews, feeling these provided an opportunity for more open communication, and the clinician to assess symptoms:

*‘When you go down there, you do get the opportunity then if there’s anything that you wish to mention … from the doctor’s point of view, he’s trying to work out a diagnosis without seeing the patient … It’s difficult.’* (Pt36)

### Patient–clinician relationship

Overall, 381 (68.5%) survey responders (and all interviewees) reported that their reviewer was a pharmacist; 77 (13.8%) were unsure. All audio-recordings were of a pharmacist reviewer. A majority (*n* = 447, 80.4%) were ‘reasonably’, ‘mostly’, or ‘very confident’ that their reviewer knew enough about them and their health (Table 3).

The odds of being satisfied with the review were strongly associated with the patient having received previous care from the reviewer (OR 3.00, 95% CI = 1.66 to 5.42) and having confidence in the reviewer (OR 16.9, 95% CI = 9.8 to 29.1) (see Supplementary Table S1). During the interviews, most participants described clinical pharmacists as experts in medication. Several considered a pharmacist-led review a good use of resources because it freed up GP time for other activities. Others described a lack of confidence in the pharmacist’s ability to enact decisions to change prescribing or expressed a preference for a GP-led review, as they perceived GPs to have greater clinical knowledge and expertise than pharmacists:

*‘… he’s not an actual doctor though, is* [he]*? … I’m not too sure how he’s got to change my medication, if he has got the power. I thought the doctor was the one who does that.’* (Pt2)

Some expressing this view described feeling reassured by pharmacists acknowledging uncertainty, and that the pharmacist had spoken with a GP before the review or would do so if necessary:

*‘She was quite open and honest to say, “Well, I’ll speak to doctor on that and we’ll get back to you”, which I thought was reassuring … They’d … done their homework prior because she’d said the doctor and herself had … reviewed my medications separately and then met up, which I thought was good.’* (Pt39)

### Seeking patient participation from the outset

Around two-thirds of survey responders agreed that the reviewer made it ‘clear that a decision needed to be made’ (*n* = 368, 66.2%) and wanted to know ‘how the patient wanted to be involved in decision making’ (*n* = 359, 64.6%) (Figure 1).

In the interviews, participants did not specifically talk about whether their pharmacists had asked how they wanted to be involved in decision making. However, audio-recordings of reviews showed that pharmacists sought patient involvement from the outset, with some using the pre-review leaflet (which practices were asked to provide to patients) as a mechanism of encouraging patient participation:

*‘The plan is for us to chat around your drugs and just to see how you’re getting on and whether you’ve got anything you would like to get out of chatting to me … I’ve got a few ideas. I’ve had a chat to* [GP name] *and we looked at your meds but I’m happy for you to drive it … how you wish.’* (Pharmacist [Ph2], audio-recording of review)

### Provision of information

Nearly four-fifths (*n* = 439, 79.0%) of survey responders reported that their reviewer was good–excellent at ‘explaining things clearly’ (Table 4). Reviewers were generally considered to have helped the patient understand the information provided during the review (*n* = 387, 69.6% agree and *n* = 116, 20.9% disagree), but there was less agreement that reviewers explained different treatment options available (*n* = 298, 53.6% agree and *n* = 202, 36.3% disagree) and the corresponding advantages and disadvantages (*n* = 324, 58.3% agree and *n* = 176, 31.7% disagree) (Figure 1).

**Table 4.** Review decision-making process (*N* = 556)^a^

Survey question	*n* (%)
**Information provision**	
Explaining things clearly (CARE instrument)	
Poor or fair	77 (13.8)
Good	119 (21.4)
Very good or excellent	320 (57.6)
**Discussion of priorities**	
Effort to listen to things that matter most to patient (CollaboRATE instrument)	
No effort	21 (3.8)
A little or some effort	165 (29.7)
A lot or every effort	334 (60.1)
Effort to include what matters to patient in decision-making (CollaboRATE instrument)	
No effort	40 (7.2)
A little or some effort	159 (28.6)
A lot or every effort	318 (57.2)
Reviewer asked what patient would like to discuss (PACIC instrument)	
No	129 (23.2)
Yes	341 (61.3)
Not sure	67 (12.1)
**Agreeing on decisions**	
Decisions to change medicines was made during review	
No	243 (43.7)
Yes	296 (53.2)
Satisfaction with changes made^b^	
Dissatisfied or very dissatisfied	23 (7.8)
Neither dissatisfied nor satisfied	48 (16.2)
Satisfied or very satisfied	222 (75.0)
**Summarising and follow-up**	
Patient provided with a copy of my treatment plan (PACIC instrument)	
No	450 (80.9)
Yes	44 (7.9)
Has a follow-up appointment been arranged?	
No	374 (67.3)
Yes	115 (20.7)
Not sure	53 (9.5)

^a^Percentages based on total survey responders; percentages do not sum to 100% owing to missing data. ^b^Percentages reported only for responders who reported a change in medication being made, *n* = 296. CARE = Consultation And Relational Empathy measure. PACIC = Patient Assessment of Chronic Illness Care measure.

Interviewees spoke mostly of wanting information about the effectiveness and need for their medication, and to better understand their medications and health. These participants welcomed the openness of the pharmacist to their questions and felt they received comprehensive and reassuring responses:

*‘Information is the thing that I got. I just thought it was a good idea to make sure … that I’m not … taking any medication that I don’t need to … also that the medications that I’m taking don’t become a cocktail in any way.’* (Pt39)

### Discussion of patient priorities

A majority of responders felt that ‘a lot’ or ‘every’ effort was made to listen to what mattered to them (*n* = 334, 60.1%), to include this within the decision-making process (*n* = 318, 57.2%), and that they had been asked what they wanted to discuss during the review (*n* = 341, 61.3%) (Table 4).

Audio-recordings of reviews showed that throughout the review, pharmacists actively sought to elicit patient preferences in relation to each medication by checking whether the medication was being taken regularly, alongside when and how, and the patient’s understanding of what it was for. Many interviewees appreciated this approach, describing feeling heard and understood, and comfortable to express concerns and preferences:

*‘She listened to me … took everything on board that I said … so the two things I had queries about we got aired at the very beginning.’* (Pt22)

Several others, however, expressed frustration when they felt the pharmacist had not fully listened or understood their concerns, and failed to consider their personal circumstances. As a result, they felt they lacked the advice and support they needed, particularly regarding alternative treatment and non-pharmacological options:

*‘I feel that they could’ve done more ... whether there was something else I could do … different that would help me … but there weren’t … none of that. It was just literally, why are you taking this … carry on with that.’* (Pt23)

### Agreement on decisions

Just over half of survey responders (*n* = 296, 53.2%) reported that the review led to a decision to change medications, with three-quarters (*n* = 222/296, 75.0%) satisfied with the change. There was no evidence (*P* = 0.90) that overall satisfaction was associated with whether or not a change had been made (see Supplementary Table S1). Around half of responders agreed that their reviewer had asked them what their preferred treatment option was and had weighed the different treatment options (*n* = 300, 54.0%) (Figure 1). Just over half (*n* = 315, 56.7%) agreed that they had selected the treatment option together with the reviewer, with around two-thirds (*n* = 384, 69.1%) reporting reaching an agreement on how to proceed.

Collaborative decision making was observed within most of the audio-recordings of reviews, which showed that following proposals to change medicines pharmacists tended to seek patient involvement using questions such as *‘how does that sound?’* (Ph2/Ph21), *‘what would you like to do?’* (Ph1/Ph19/Ph14/Ph10), or *‘shall we see how that goes?’* (Ph3).

Many interviewees valued the opportunity to discuss the pros and cons of medication changes and fully participate in decisions, including whether to continue or modify their current treatment regimen:

*‘I’m always open to discussion and debate, which is … why I’m going to go onto this new type of statin which* [the pharmacist] *thinks is more effective. You listen and learn … It’s a two-way conversation all the time.’* (Pt25)

A few, however, conveyed a preference for expert-driven decision making and did not feel the need to ask questions as they viewed their reviewer as the *‘expert’* (Pt27).

When a medication change was suggested by the pharmacist, interviewees spoke of needing to fully understand the reasons behind the proposal before accepting it. Those who accepted a therapeutic change felt reassured that they would receive ongoing support from the pharmacist and could reverse the decision later if they wished:

*‘I could have said no. She asked me, not told me. She said can I reduce* [esomeprazole] *… and try it out for a month? But I don’t mind … if they try it out, because sometimes you get used to a medicine and it don’t do you any good anyway.’* (Pt28)

Those who did not want to make changes to their current treatment regimen felt confident rejecting the pharmacist-recommended changes because they saw no need for adjustments or feared changes might disrupt their health or routine. Audio-recordings showed that when patients declined these changes, pharmacists respected their decision and recommended revisiting this discussion later:

*‘We’re going to reduce the* [zopiclone quantity] *but we’re not reducing the dose … and we just made a note there that we’ll discuss the zopiclone again in the future.’* (Ph3, audio-recording of review)

### Summary provision and follow-up arrangements

Only a small minority of survey responders reported receiving a written treatment plan (*n* = 44, 7.9%) or having a follow-up appointment arranged (*n* = 115, 20.7%) (Table 4). Although few interviewees mentioned receiving a treatment plan, a verbal summary of next steps, including discussion of follow-up, was observed within most of the recorded reviews. Additionally, audio-recordings demonstrated that pharmacists checked patient understanding and reaffirmed decision making before concluding the review:

*‘We’ve come up with ... an action plan ... You told me that you weren’t getting on* [with drug brand] *… We talked about* [alternative dosing strategy] *… We checked your blood pressure and it’s lovely* [and have decided to stop one drug and arrange further monitoring] *... we’ll meet again or perhaps do it over the phone, if that’s alright?’* (Ph4, audio-recording of review)

Several interviewees talked about planned next steps for implementing medication changes and/or monitoring progress, and described feeling reassured by the ongoing support offered by the pharmacist:

*‘She made a note for me and gave it to me at the end sort of saying … what we discussed like taking my blood pressure, not taking that tablet … I’m gonna get a follow-up … she’s going to phone me in three weeks … to see how my blood pressure is. I think that is quite impressive.’* (Pt10)

## Discussion

### Summary

Overall, survey feedback reflected high levels of patient satisfaction that was positively associated with perceptions of shared decision making. In general, key elements of shared decision making were evident in all three data sources, although some participants expressed frustration that their concerns were not fully addressed. Patients described feeling comfortable to decline pharmacist-recommended changes, and pharmacists respected patient autonomy in doing so. A desire to avoid disruption, or lack of compelling justification for change, were key reasons cited for declining reviewers’ recommendations.

Patient preparation for the review was key to the value patients placed on it and patients’ engagement; practices’ failure to implement the pre-review leaflet as intended had a negative impact on patient experience. Reviews delivered by telephone were generally considered acceptable, albeit with some disadvantages.

Delivery of reviews by pharmacists was generally well received, despite patients often not knowing the clinician and concerns about pharmacists’ authority to alter prescribing; prior discussion between pharmacist and GP was considered reassuring, and familiarity with the clinician was valued. Although verbal summaries of reviews were provided, written treatment plans were not usually given to patients and follow-up was infrequent.

### Strengths and limitations

The mixed-methods approach and triangulation of different datasets provided in-depth understanding of patient experience and satisfaction with the IMPPP medication review intervention. The findings provide valuable insights pertinent to intervention implementation, independent of the trial effectiveness outcomes. However, several factors might limit generalisability of the findings.

The survey response rate was high at 72.5%, but findings may suffer from response bias if individuals with negative experiences were less likely to participate. Additionally, individuals who declined or did not attend the patient-facing review appointments may differ systematically from those included in the survey.

Survey responders and interviewees predominantly identified as White British with lower levels of socioeconomic deprivation, meaning that diverse demographic and cultural perspectives were not fully represented. Furthermore, the data were gathered as part of a process evaluation in a trial of a complex intervention comprising a four-step medication review process, and not within usual general practice. Although interviewees commented on the convenience of telephone consultations, data on mode of delivery were incomplete and potentially unreliable, so a formal analysis of its association with satisfaction was not conducted. As with all interview studies, it is not possible to fully exclude bias introduced by the interview process itself. However, efforts were made to minimise this risk (for example, experienced interviewers, standardised interview protocols, and dual-coding of transcripts) and we are confident the insights obtained are authentic.

### Comparison with existing literature

A recent review of telehealth medication reviews shows high levels of patient satisfaction, suggesting these services are feasible, save costs, and improve care.^19^ In the current study, satisfaction with largely pharmacist-led reviews, which were mostly conducted via telephone, were similarly high, suggesting that patient needs and expectations were met. Nevertheless, limitations of telephone consultations are well recognised^20^ and this was reflected in the current study where, despite acknowledging the convenience of telephone consultation, some patients noted the lost opportunity for more open and comprehensive assessment.

Structured medication reviews are a central element of UK medicines optimisation policy, but patients do not always experience these reviews as patient centred.^21^ We have shown that reviews delivered in the context of support to deliver patient-centred care (such as relevant clinician training and pre-review preparation) are perceived positively and as patient centred by patients.

Patients in the current study were more satisfied when they experienced shared decision making, and valued being adequately informed, prepared for the interaction, and receiving patient-centred communication incorporating their priorities and preferences. This aligns with a recent scoping review showing considerable variation in the level of information patients desire about their medications, underlying the need to personalise communication and information during medication reviews.^22^ Moreover, inclusion of patients' personal priorities and preferences in care enhances effective patient–practitioner communication making patients feel heard, understood, respected, and involved in their treatment.^23^ This leads to increased confidence in treatment plans, greater satisfaction with care, and higher levels of trust in healthcare providers.^5,24–27^

### Implications for practice

Within the broader strategy of medicines optimisation, medication reviews are one of several approaches aimed at ensuring that medicines use is safe, effective, and aligned with patient priorities, although evidence of clinical benefit remains limited.^28–30^ Existing guidelines for structured medication review in England emphasise that reviews should be conducted by trained clinicians working within their scope of practice using the principles of shared decision making to underpin conversations with patients.^3,31,32^ Our findings suggest that the IMPPP model of medication review delivery helps achieve patient-centred care, shared decision making, and effective communication, and thus has the potential to improve experience of care and meet policy objectives.

Our findings point to the importance of patients having confidence in their clinician, yet a substantial minority of patients report this is lacking and interviews additionally pointed towards uncertainty regarding pharmacists’ clinical authority. Improving patient awareness about the role of the growing clinical pharmacist workforce may thus lead to better patient experience, and is to be encouraged. How this should be achieved remains uncertain, although our results suggest improved collaborative working between GPs and pharmacists may be one approach of value.

To enhance patient experience and satisfaction, it is also important that shared decision-making principles are integrated throughout the review process. The current study shows patients value being properly prepared but also suggests that the conclusion of reviews is suboptimal, lacking written action plans or clear follow-up. Practice-level policies should therefore include standards for sharing of pre-review information and providing post-review written summaries and follow-up. Pre-review notifications should detail the reviewer’s scope of practice and the review agenda, and prompt patients to reflect on their medications and identify what they want to discuss. Post-review summaries should clearly document agreed modifications, therapeutic goals, and guidance for monitoring treatment effectiveness, managing issues, and attending follow-up appointments.

By taking these issues into consideration, medication review policy and practice has the potential to improve shared decision making and patient satisfaction, empowering patients to more actively engage in their review and enhancing patient experience.

## Data

All data requests should be submitted to the corresponding author for consideration. Access to anonymised data may be granted following review via the University of Bristol Research Data Storage Facility.
